# Preferential retention of algal carbon in benthic invertebrates: Stable isotope and fatty acid evidence from an outdoor flume experiment

**DOI:** 10.1111/fwb.13492

**Published:** 2020-03-02

**Authors:** Thomas Kühmayer, Fen Guo, Nadine Ebm, Tom J. Battin, Michael T. Brett, Stuart E. Bunn, Brian Fry, Martin J. Kainz

**Affiliations:** ^1^ Department of Limnology and Oceanography University of Vienna Wien Austria; ^2^ WasserCluster Lunz – Inter‐University Center for Aquatic Ecosystems Research Lunz am See Austria; ^3^ Simon F.S. Li Marine Science Laboratory School of Life Sciences The Chinese University of Hong Kong Hong Kong China; ^4^ Stream Biofilm and Ecosystem Research Laboratory École Polytechnique Fédérale de Lausanne Lausanne Switzerland; ^5^ Department of Civil Engineering University of Washington Seattle WA USA; ^6^ Australian Rivers Institute Griffith University Brisbane Qld Australia

**Keywords:** compound‐specific stable isotopes, food quality, food webs, headwaters, River Continuum Concept

## Abstract

1. According to the River Continuum Concept, headwater streams are richer in allochthonous (e.g. terrestrial leaves) than autochthonous (e.g. algae) sources of organic matter for consumers. However, compared to algae, leaf litter is of lower food quality, particularly ω‐3 polyunsaturated fatty acids (n‐3 PUFA), and would constrain the somatic growth, maintenance, and reproduction of stream invertebrates. It may be thus assumed that shredders, such as *Gammarus*, receive lower quality diets than grazers, e.g. *Ecdyonurus*, that typically feed on algae.
2. The objective of this study was to assess the provision of dietary PUFA from leaf litter and algae to the shredder *Gammarus* and the grazer *Ecdyonurus*. Three different diets (algae, terrestrial leaves, and an algae–leaf litter mix) were supplied to these macroinvertebrates in a flume experiment for 2 weeks. To differentiate how diet sources were retained in these consumers, algae were isotopically labelled with ^13^C.
3. Both consumers became enriched with ^13^C in all treatments, demonstrating that both assimilated algae. For *Gammarus*, n‐3 PUFA increased, whereas n‐6 PUFA stayed constant. By contrast, the n‐3 PUFA content of *Ecdyonurus* decreased as a consequence of declining algal supply.
4. Results from compound‐specific stable isotope analysis provided evidence that the long‐chain n‐3 PUFA eicosapentaenoic acid (EPA) in both consumers was more enriched in ^13^C than the short‐chain n‐3 PUFA α‐linolenic acid, suggesting that EPA was taken up directly from algae and not from heterotrophic biofilms on leaf litter. Both consumers depended on algae as their carbon and EPA source and retained their EPA from high‐quality algae.

## 1 INTRODUCTION

In their seminal work, Vannote, Minshall, Cummins, Sedell, and Cushing (1980) introduced the River Continuum Concept (RCC), suggesting that riparian vegetation in forested headwater streams limits autotrophic production by shading and supplies considerable amounts of allochthonous organic matter (OM). It has often been assumed that benthic invertebrates in headwater streams are dependent on this allochthonous OM as dietary energy, an assumption that has been recently questioned (Lau, Leung, & Dudgeon, 2008b; Rasmussen, 2010; Torres‐Ruiz, Wehr, & Perrone, 2007). There is still uncertainty as to whether terrestrial leaves can provide sufficient dietary energy and resources to support consumer fitness and answering this question requires detailed information on the dietary quality of autochthonous and allochthonous OM for invertebrate consumers in streams.

Food sources differ in their nutritional quality in stream ecosystems (Brett et al., 2017; Guo et al., 2018; Lau, Leung, & Dudgeon, 2008a). For aquatic consumers, terrestrial leaves are considered as a low‐quality resource (Allan & Castillo, 2007) because they contain a high content of recalcitrant OM, such as cellulose, lignin, and hemicelluloses (Meyers & Ishiwatari, 1993), and short‐chain polyunsaturated fatty acids (PUFA), e.g. linoleic acid (LIN; 18:2n‐6) and α‐linolenic acid (ALA; 18:3n‐3; Torres‐Ruiz et al., 2007). They are also deficient in high‐quality dietary nutrients, such as long‐chain PUFA, in particular eicosapentaenoic acid (EPA; 20:5n‐3) and docosahexaenoic acid (DHA; 22:6n‐3; Lau, Leung, & Dudgeon, 2009). After leaves enter stream channels, fungi and bacteria colonising leaves can increase their nutritional quality in terms of lower C:N ratios and increased protein content (Allan & Castillo, 2007). However, fungi and bacteria also lack long‐chain PUFA, including EPA and DHA, that support somatic growth and reproduction of invertebrates (Guo, Kainz, Sheldon, & Bunn, 2016a; Lau et al., 2009). Studies on terrestrial and marine fungi reported that 16:0, 18:0, 18:1n‐9, and LIN are the most common fatty acids (FA) in fungi (Cooney, Doolittle, Grahl‐Nielsen, Haaland, & Kirk, 1993; Stahl & Klug, 1996). Bacteria contain 15:0, 17:0 and their branched derivatives, as well as vaccenic acid (18:1n7; Desvilettes, Bourdier, Amblard, & Barth, 1997), but generally lack ω‐3 PUFA.

In contrast, algae are of higher nutritional quality with more easily accessible carbon, in particular the long‐chain PUFA: arachidonic acid (ARA, 20:4n6), EPA, and DHA; it is therefore expected that aquatic consumers will derive high‐quality carbon and essential biomolecules from algae (Brett et al., 2017). Algal PUFA, especially the long‐chain n‐3 PUFA EPA and DHA, enhance the somatic growth and reproduction of aquatic consumers, yet algal taxa differ markedly in their FA composition and thus in their nutritional quality. For example, diatoms contain EPA and DHA (Brett & Müller‐Navarra, 1997), whereas cyanobacteria lack these PUFA as well as nutritionally important sterols (Martin‐Creuzburg, Von Elert, & Hoffmann, 2008). However, most aquatic consumers have a limited ability to convert the short‐chain PUFA LIN or ALA to long‐chain PUFA (Brett & Müller‐Navarra, 1997). The physiological mechanism for such conversion is still poorly studied for most invertebrates (Guo, Bunn, Brett, & Kainz, 2017; Torres‐Ruiz et al., 2007).

The two long‐chain PUFA most often shown as essential for stream macroinvertebrates are EPA and ARA, whereas DHA is generally lacking (Guo et al., 2018). Eicosapentaenoic acid plays a critical role for the reproduction and emergence of insects (Stanley‐Samuelson, 1994) and ARA for somatic growth and reproduction (Ahlgren, Vrede, & Goedkoop, 2009 for chironomids and ephemerids). Although DHA is not retained in most stream macroinvertebrates, high DHA contents generally found in stream fish (Heissenberger, Watzke, & Kainz, 2010) require bioconversion from dietary precursors, such as EPA, to DHA in their hepatocytes (Murray, Hager, Tocher, & Kainz, 2014). For macroinvertebrates, the consumption of algae that contain EPA leads to a higher growth rate and reproduction in shredders (Guo, Kainz, Valdez, Sheldon, & Bunn, 2016c) and grazers (Guo et al., 2016a). Algal biofilms growing on leaf surface can increase the nutritional quality for consumers (Findlay, 2010; Guo et al., 2018) and support their somatic growth (Manning et al., 2015). Such biofilms can be considered as *peanut butter on crackers* (Cummins, 1974), emphasising that only part of the ingested diet is of high nutritional value.

Fatty acids are also used as biomarkers of dietary bacterial, algal, and terrestrial sources in aquatic organisms (Jardine et al., 2015; Kainz & Mazumder, 2005). As such, the FA composition of diets and consumers can be used to assess how similar the FA retained in consumers are to dietary FA. Bulk stable isotopes (Perga, Kainz, Matthews, & Mazumder, 2006) and compound‐specific stable isotopes (Taipale, Kainz, & Brett, 2011) used in combination with FA can yield more detailed information about trophic transfer for OM sources. Although there is increasing evidence that algae FA are more highly retained in aquatic consumers than terrestrial FA (Brett, Kainz, Taipale, & Hari, 2009; Guo, Kainz, Sheldon, & Bunn, 2016b; Torres‐Ruiz et al., 2007), it remains to be elucidated if aquatic macroinvertebrates can convert dietary FA from either algae or terrestrial sources to build their somatic biomass. For example, if headwater consumers mainly feed on terrestrial leaves, as suggested by the RCC, does carbon from allochthonous sources make an important contribution to the tissues of headwater stream invertebrates?

Based on such uncertainties, we designed an outdoor flume experiment to assess the provision of dietary PUFA from leaf litter (allochthonous source) and algae (autochthonous source) to stream macroinvertebrates using an isotopic tracer. Algae were labelled with ^13^C to differentiate the dietary uptake of these two sources in consumers. By artificially manipulating the abundance of the heavy carbon isotope (^13^C), differences in uptake by different producers (e.g. fast uptake by microalgae, but slow uptake by terrestrial leaves due to difference in tissue turnover rates) were thought to result in differential labelling of the producers, thereby allowing to track specific food sources when the consumers are analysed at predetermined times after enrichment. We tested the hypotheses that: (1) invertebrates from different feeding guilds obtain most of their carbon from algae and not from leaf litter; and (2) long‐chain PUFA in both consumers are derived directly from algae and not converted from precursors.

## 2 METHODS

### 2.1 Flume experiment

This study was performed in an outdoor experiment, consisting of 18 flumes, for 14 days (11–25 July 2017) at WasserCluster Lunz (Austria). Two feeding guilds were used representing a common shredder (*Gammarus *sp., an amphipod) and a common grazer (*Ecdyonurus *sp., a heptageniid mayfly), and all fed with three different diets: (1) naturally growing stream algae (treatment A); (2) leaf litter (treatment L); or (3) mixed diet, i.e. algae and leaf litter (treatment M), with six replicated flumes for each diet. The flumes were composed of acrylic plastic (300 cm long, 5 cm width, and 10 cm high) and their upper end was closed, whereas the lower end was open to allow stream water to freely flow out. The flumes were covered with a light transparent sheet to avoid rainwater or leaves directly entering the flumes. The flume water was supplied from a nearby stream (Lunzer Seebach) and pumped into each of the flumes at a constant supply rate (0.1 L/s), creating an average velocity of 0.1 m/s. In each flume, two stainless steel nets (5.5 × 11 cm) were installed; one 50 cm downstream of the flume inflow and the other 100 cm before the outflow to prevent invertebrates from escaping due to drift.

The experimental stretch of the flumes between the nets was covered with unglazed ceramic tiles (5 × 5 × 0.5 cm) to allow biofilm development. Twenty‐seven days before the start of the experiment, the flumes were supplied with unfiltered stream water and algae from the stream water formed biofilms on the tiles; the leaf litter treatment was not covered with tiles over the first 27 days to limit algae growth. No algal growth was observed at the channel walls.

Leaf litter from beech (*Fagus sylvatica*; a common tree species around the study area) was collected from nearby streams and added to the flumes 1 day before the start of the experiment to treatments L and M. Leaf litter was conditioned for 1 week in the dark to minimise photosynthetic biofilm growth, but some periphytic taxa may have survived on leaf litter without light. Six stones from the adjacent stream were added, and regularly brushed from algae, to each flume to better simulate stream conditions and offer hiding places for invertebrates.

The macroinvertebrates *Gammarus* and *Ecdyonurus* were collected 1 day before the start of the experiment from a nearby stream (Lunzer Seebach). The genus *Gammarus* is represented by the single species *Gammarus fossarum* (Koch, 1835) in these streams. Several species of *Ecdyonurus* are present within the studied headwater stream, including *Ecdyonurus* *dispar* (Curtis, 1834), *Ecdyonurus* *helveticus* (Eaton, 1885), *Ecdyonurus* *picteti* (Meyer‐Dür, 1864), and *Ecdyonurus* *venosus* (Fabricius, 1775) (J. Waringer, personal communication). The *Gammarus* and *Ecdyonurus* were separated into different flumes to avoid food competition and 20 individuals added to each, resulting in three replicates per treatment per taxon.

### 2.2 Flume labelling

To distinguish dietary carbon from leaf litter and algae in the invertebrates, all treatments were enriched in ^13^C by labelling the flumes with ^13^C enriched NaHCO_3_. In experiment L, algae were carefully brushed from leaf litter every second day to minimise dietary contribution of ^13^C‐labelled periphytic lipids. Previous studies showed that the addition of ^13^C enriched NaHCO_3_ did not label plant stems, so leaf litter carbon would isotopically retain its natural background abundance (Maddi, Carman, Fry, & Wissel, 2006). This isotopic tracer was first added 12 days before the start of the experiment and then continuously supplied every second day until completion. For this tracer addition, the water flow was blocked off for 1 hr (always between 10 and 11 a.m., during which time the photosynthetic productivity was assumed to be high), but water temperature did not increase considerably. Ten millilitres of 0.74 g/L^ 13^C‐labelled NaHCO_3_ solution was added to each of the A and M treatment flumes to double the ^13^C content from 0.029 to 0.057 mM. This increased the delta (δ^13^C) values of the water to approximately +980‰. However, because the labelling was only 1 hr every second day and algae also fixed carbon during the remainder of the day (additional 15 hr in July), the δ^13^C value of the algae was expected to be around +20‰. Nevertheless, photosynthetic efficiency naturally differs during daytime, weather conditions and physiological variability of the algae. Thus, the realised δ^13^C values were expected to differ somewhat from the expected +20‰ target. Data loggers (HOBO™) were used to log light and temperature every hour during the experiment.

### 2.3 Sample collection

At the beginning (T1) and the end of the experiment (T2), algae samples for FA and carbon content measurements were collected from each flume. Additionally, three random samples were taken after 1 week (T1.5). From each flume, one randomly chosen tile covered with algae was scraped and immediately cooled and frozen at −20°C. For the determination of FA and carbon of leaf litter, three replicate leaf samples from each flume were taken at the start and at the end of the feeding experiment, respectively. Leaf materials were stored at −20°C. At the end of the experiment, all living invertebrates were collected from the flumes, frozen at −80°C and subsequently freeze‐dried (Virtis™ Genesis Freeze Dryer) for 2 days and then ground and homogenised using a pestle and mortar.

### 2.4 Algal chlorophyll‐a

Chlorophyll‐*a* (Chl‐*a*) was measured for algal biomass assessment on the tiles and on leaf litter prior to and after the experiment. From each flume, biofilm from randomly picked tiles or leaves was scraped, filtered on a Whatman GF/F (25 mm diameter) filter. The Chl‐*a* was then extracted using acetone (90%) and subsequently measured on a fluorescence spectrophotometer (Hitachi F‐7000).

### 2.5 Algal community composition

Randomly chosen tiles from the flumes were scraped to determine the autotrophic community composition. Samples were diluted using MQ‐water (15 ml) and fixed with Lugol solution (1:50 dilution). The autotrophic community composition was determined and counted using an inverted microscope (Nikon™ Eclipse TS 100; objective 20×).

### 2.6 Invertebrate somatic growth assessment

The initial dry weight of each consumer that was put into the flumes was estimated by a regression equation between body dry weight and body length, and/or wet weight. For *Gammarus*, the initial wet and dry weights of 38 individuals were measured, and the following regression equation was established; i.e. dry weight (*y*) = 0.1313 times body length (*x*) + 1.151, *r* ^2^ = 0.81. The regression equation between *Ecdyonuru*s body length (head to end of abdomen in mm) and dry weight (mg) was established by measuring 44 individuals, i.e. dry weight (*y*) = 2.6104 times body length (*x*) − 16.808, *r* ^2^ = 0.88.

For the 20 individuals that were placed in each flume, initial wet weight or body length was measured and a mean for the 20 individuals was calculated. Based on these data, we calculated the starting mean dry weight per individual for each flume (3.18 mg for *Gammarus*, 6.05 mg for *Ecdyonurus*).

After 14 days, all living invertebrates (67% *Gammarus* and 76% *Ecdyonurus* survived) from each flume were collected and freeze‐dried, weighed, and divided by the number of remaining invertebrates to determine the mean dry weight per individual at the end of the experiment. The difference between the start and end point was the increase in dry weight over 2 weeks. The difference divided by the initial dry weight was the somatic growth per mg initial weight (Crenier et al., 2017). Somatic growth was expressed as gain or loss of weight (in %) relative to the initial weight.

### 2.7 Fatty acid analysis

All samples were freeze‐dried (Virtis Genesis Freeze Dryer) and homogenised before lipid and FA analysis. Lipids from algae (*c. *20 mg) and leaf litter (*c. *150 mg) were extracted and methylated according to the methods reported elsewhere (Guo et al., 2016c). We used nonadecanoic acid (19:0) as an internal standard. In brief, fatty acid methyl esters (FAME) were analysed using a gas chromatograph (THERMO Trace; FID 260°C, Carrier gas: He: 1 ml/min, Detector gases: H_2_: 40 ml/min, N_2_: 45 ml/min, air: 450 ml/min, temperature ramp: 140°C (5 min) − 4°C/min–240°C (20 min) = 50 min) equipped with a temperature‐programmable injector and an autosampler. FAME were separated by a Supelco™ SP‐2560 column (100 m, 0.25 mm i.d., 0.2 µm film thickness), identified by comparison of their retention times with known standards (37‐component FAME Mix, Supelco 47885‐U; Bacterial Acid Methyl Ester Mix, Supelco 47080‐U) and quantified with reference to seven‐point calibration curves based on known standard concentrations. The retention of FA in consumers is defined as the ability of organisms to regulate ingested FA detected in their tissues (Kainz, Arts, & Mazumder, 2004).

### 2.8 Bulk stable isotope analysis

Freeze‐dried leaf litter (600 ± 30 μg) and algae or invertebrate samples (300 ± 15 μg) were weighed in tin capsules (duplicates) for subsequent carbon determination, and their stable isotopes (δ^13^C) values were quantified using an A Flash HT Plus CNSOH elemental analyser interfaced with a Conflo IV device (Thermo, Bremen, Germany) to a continuous flow stable isotope ratio mass spectrometer (Delta V Advantage IRMS). Our reference gas was CO_2_ (messergroup.com). The mass spectrometer was calibrated to the at‐air international standards using CH7 and USGS24 for carbon. All stable isotope values were reported in the δ notation where δ^13^C = ([R_sample_/R_standard_] − 1) × 1,000, and R is ^13^C:^12^C.

### 2.9 Compound‐specific stable isotope analyses

Compound‐specific stable isotope analyses were used to assess the origin and isotopic composition of different FA molecules. Fatty acids were separated using a gas chromatograph (Trace 1310 Thermo™) with helium as the carrier gas. The GC was coupled to an Isolink 2 where the separated FA where combusted at 1,000°C. The generated CO_2_ was transported with the carrier gas (helium) to the Conflo IV where each sample was diluted with the carrier gas helium and connected with the reference CO_2_ gas. Finally, all CO_2_ molecules were analysed in the IRMS (Delta V Advantage).

### 2.10 Data analysis

All statistical analyses were performed using R (R Studio 0.99.902). The significant level for all tests was set at 0.05. A Student *t* test or Mann–Whitney *U* test was used to compare paired samples, depending on the distribution of the data. An ANOVA or Kruskal–Wallis *H*‐test was applied to compare multiple samples. Principal components analysis was conducted to characterise the differences in FA compositions between algae, leaf litter, and macroinvertebrates, using the *vegan* package in R on arcsin‐transformed FA values (%). The level of statistical significance for all tests was set at *p* < 0.05.

## 3 RESULTS

### 3.1 Algal biomass

Algal biomass, assessed by its Chl‐*a* content, was significantly greater on tiles than on leaf litter (*p* < 0.001, *t* test). The Chl‐*a* content on the tiles decreased, but not significantly, over time from 7.7 to 4.3 µg Chl‐*a*/cm^2^ (*p* = 0.057, *t* test) because of an uncontrollable influx of organic particles from the stream water entered the flumes. The Chl‐*a* content on the leaf litter also increased but not significantly throughout the experiment, from 0.04 to 0.07 µg Chl‐*a*/cm^2^ (*p* = 0.059, *t* test).

### 3.2 Algal community composition

The algal density decreased significantly during the experiment from *c. *3.6 × 10^5^ to *c. *2.1 × 10^5^ cells/cm (*p* = 0.023, *t* test). Diatoms (mostly composed of the genera *Achnanthidium*,* Diatoma*, and *Cymbella*) were the most abundant group (>90% of identified cells), followed by chlorophytes (mostly *Chlamydomonas* and *Pediastrum*; <10%) and cyanobacteria (<1%). Although the relative abundance of diatoms increased, the absolute abundance of diatoms and chlorophytes decreased significantly (*p* < 0.05, *t* test).

### 3.3 Invertebrate somatic growth

No significant somatic growth for both consumers was observed in any of the three treatments after the feeding experiment. For *Gammarus*, the overall body mass increase was 28.3% in treatment A, 21.2% in treatment L, and 21.8% in treatment M, but was not significant among the three diet treatments (*p* = 0.695, ANOVA). Somatic growth in *Ecdyonurus* also did not differ significantly among treatments (*p* = 0.442, ANOVA), but in contrast was −15.4% in treatment L, −13.6% in treatment A and −2.85 in treatment M.

### 3.4 Fatty acid variation

**Figure 1:**
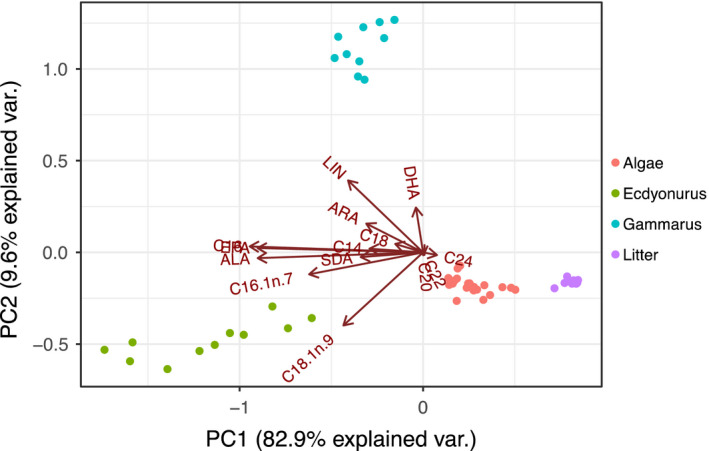
Principal components analysis of arcsin‐transformed fatty acids (%) of algae, leaf litter, and *Ecdyonurus* and *Gammarus* before and after the feeding experiment. See text for fatty acid abbreviations; the vectors of 16:0 and eicosapentaenoic acid overlap

Fatty acid patterns in diets and consumers (*Gammarus* and *Ecdyonurus*) showed clear separation from each other (Figure 1). Algae and leaf litter were associated with long‐chain, saturated FA (22:0 and 24:0) and had very low variation in their FA content before and after the experiment. The FA composition of *Gammarus* differed from *Ecdyonurus* mainly by LIN and DHA, whereas *Ecdyonurus* differed mainly by EPA, ALA, and typical diatom FA, such as 16:1n‐7 (palmitoleic acid).

Algae had on average 7× higher n‐3 PUFA, 5× higher n‐6 PUFA, and 2× higher total lipid contents than leaf litter (i.e. 4.4 versus 0.6 mg n‐3 PUFA g/dry weight; 1.4 versus 0.2 mg n‐6 PUFA g/dry weight; and 36 versus 18 mg total lipids g/dry weight, respectively). However, the algal FA content changed significantly over time. LIN and EPA were higher at T1.5 (*p* < 0.01, ANOVA; *H*‐test: *p* = 0.023, respectively) compared to T1 and T2. ALA, DHA, and n‐6 PUFA decreased after T1.5 (*p* < 0.001, ANOVA; *p* < 0.001, *H*‐test; *p* < 0.001, ANOVA; respectively). Omega‐3 and total PUFA increased from T1 to T1.5 (*p* < 0.001, ANOVA), but decreased from T1.5 to T2 (*p* < 0.01, ANOVA). Arachidonic acid did not change significantly, but slightly increased at T1.5. Total lipids decreased significantly from T1 to T2 (*p* = 0.017, H‐test). In contrast, the FA content in leaf litter hardly changed over time, whereas EPA and the n‐3/n‐6 ratio differed significantly during the experiment (*U* tests: *p* < 0.01).

**Figure 2:**
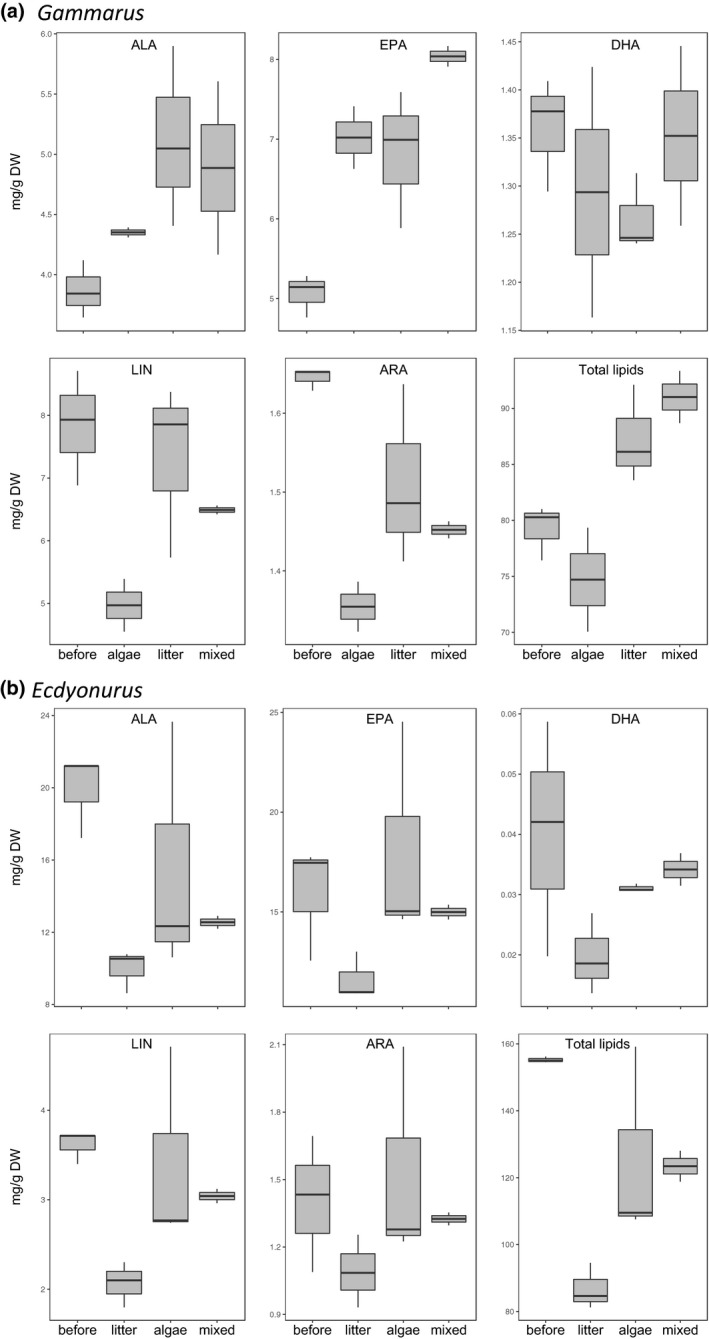
Total lipids and fatty acids in (a) *Gammarus* and (b) *Ecdyonurus* before the feeding experiment and after feeding on algae, leaf litter, or, mixed diet. ALA: alpha‐linolenic acid (18:3n‐3); EPA: eicosapentaenoic acid (20:5n‐3); DHA: docosahexaenoic acid (22:6n‐3); LIN: linoleic acid (18:2n‐6); ARA: arachidonic acid (20:4n‐6).

The total lipid and FA content in *Gammarus* and *Ecdyonurus* differed among the three treatments (Figure 2). In *Gammarus*, total lipids were highest in treatment *M* (91 ± 3 mg/g dry weight), followed by treatment L (87 ± 4 mg/g dry weight) and treatment A (79 ± 2 mg/g dry weight). *Gammarus* contained lower n‐6 PUFA (LIN and ARA) in treatment A compared to those at the beginning of the experiment (Figure 2a). Omega‐6 PUFA were higher in *Gammarus* that fed on litter and mixed diets relative to treatment A. The essential n‐3 PUFA, ALA, was higher in *Gammarus* in all diet treatments relative to *Gammarus* from the stream. Similarly, EPA was significantly higher in *Gammarus* feeding on algae, litter, and the mixed diet compared to *Gammarus* from the stream. Moreover, of all PUFA, the EPA content was most highly retained in *Gammarus*, whereas only very little DHA was detected. The n‐3/n‐6 ratio was lowest in *Gammarus* from the stream (1.1 ± 2.5) and highest in treatment A (2 ± 0.1).

In *Ecdyonurus*, however, total lipids and the FA contents did not differ significantly among different treatments, although total lipids, ω‐6 (LIN and ARA) and ω‐3 PUFA (ALA, EPA, and DHA) decreased during the experiment relative to lipids and FA in *Ecdyonurus* at the beginning of the experiment (Figure 2b). In contrast to *Gammarus*, the n‐3/n‐6 ratios in *Ecdyonurus* were much higher and ranged between treatment *M* (6.0 ± 0.1) and treatment L (6.6 ± 0.13).

### 3.5 Bulk stable carbon isotopes

**Figure 3:**
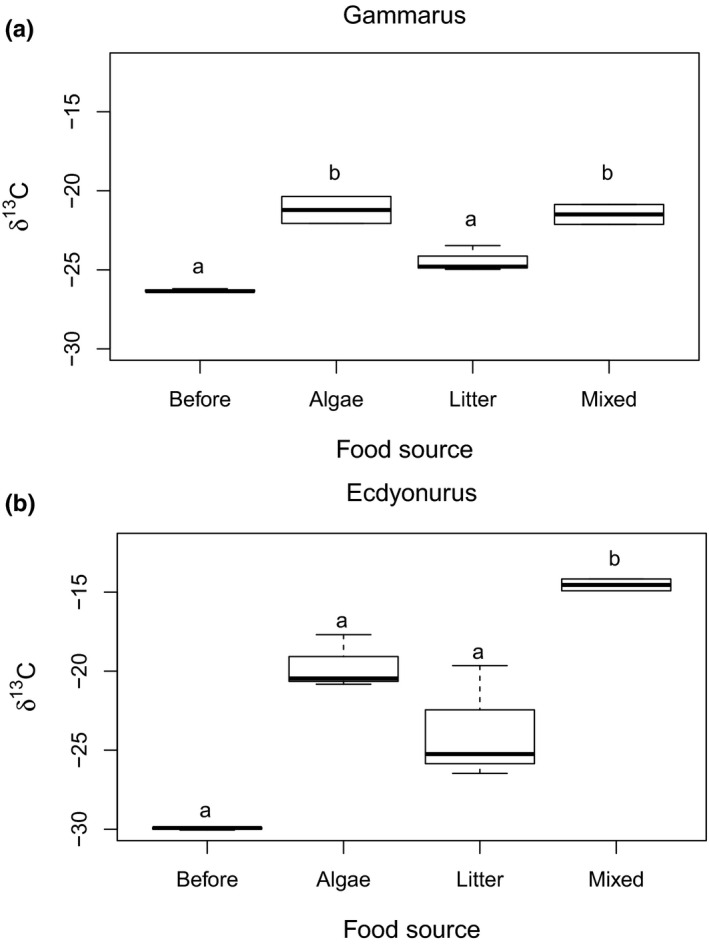
Delta ^13^C values in (a) *Gammarus* and (b) *Ecdyonurus* before the feeding experiment and after feeding on algae, leaf litter, or, mixed diet

Algal δ^13^C values varied over the duration of the experiment from −21.0‰ to +34.0‰, whereas the δ^13^C values of the litter material remained relatively constant (−30.2 ± 0.6‰, mean ± *SD*). The δ^13^C values of *Gammarus* were enriched in all treatments compared to the start of the experiment (Figure 3). *Gammarus* in the treatments A (−21.2 ± 1.2‰) and *M* (−21.5 ± 0.9‰) were significantly enriched in ^13^C compared to *Gammarus* from the nearby stream (starting conditions, −26.3 ± 0.1‰) or treatment L (−24.4 ± 0.8‰; *p* < 0.001, ANOVA; Figure 3a). The δ^13^C values of *Ecdyonurus* were enriched, but not significantly different from the start of the experiment (−29.9 ± 0.1‰), except in treatment *M* (−14.5 ± 0.5‰; *p* = 0.035, *H*‐test). *Ecdyonurus* in treatment L were isotopically lighter (−23.8 ± 3.6‰) than in the labelled treatment A (−19.7 ± 1.7‰; Figure 3b).

### 3.6 Compound‐specific stable isotope analyses

Three PUFA (LIN, ALA, and EPA) in algae were isotopically enriched (resulting in positive δ^13^C values) during the experiment and could thus be distinguished from the more negative δ^13^C values in the leaf litter (Table 1). Although periphyton was removed from leaf litter (see above), the PUFA on leaf litter were isotopically enriched, but not as strongly as the PUFA from treatment A, except for EPA.

**Table 1:** Compound‐specific stable isotope values (mean δ^13^C values; ‰) of linoleic (LIN), α‐linolenic (ALA), and eicosapentaenoic acids (EPA) in algae and leaf litter before and after the experiment, and in the grazer *Ecdyonurus* and the shredder *Gammarus* before and after feeding on algae (A), leaf litter (L), or the mixed diet (M; algae + leaf litter); Δ = difference in δ^13^C values (‰) of LIN, ALA, and EPA between before and after the experiment

	LIN			ALA			EPA		
	Before	After	Δ	Before	After	Δ	Before	After	Δ
Algae	5.7	6.8	1.1	10.27	6.25	−4.0	−9.36	14.73	24.1
Leaf litter	−37.4	−28.8	8.6	−39.2	−34.7	4.5	−40.3	8.5	48.8
*Ecdyonurus* A	−36.0	−24.4	11.6	−40.6	−36.7	3.9	−34.9	−18.4	16.5
*Ecdyonurus* L	−36.0	−27.2	8.8	−40.6	−36.7	3.9	−34.9	−25.5	9.4
*Ecdyonurus* M	−36.0	−24.2	11.8	−40.6	−34.7	5.9	−34.9	−12.1	22.8
*Gammarus* A	−33.4	−30.4	3.0	−37.7	−23.5	14.2	−36.1	−25.7	10.4
*Gammarus* L	−33.4	−33.1	0.3	−37.7	−33.7	4.0	−36.1	−31.1	5.0
*Gammarus* M	−33.4	−29.8	3.6	−37.7	−29.6	8.2	−36.1	−24.7	11.4

In both consumers, LIN, ALA, and EPA were enriched in ^13^C in each treatment. At the end of the experiment, δ^13^C_EPA_ values in *Ecdyonurus* and *Gammarus* were more enriched relative to the start of the experiment (Δ = 16.5 and 8.9‰, respectively). Compared to δ^13^C_EPA_, δ^13^C_ALA_ (Δ = 4.6 and 8.8‰, respectively), δ^13^C_LIN_ values (Δ = 10.7 and 2.3‰, respectively) were less enriched in both consumers. For *Ecdyonurus*, δ^13^C_ALA_ values were more depleted compared to δ^13^C_LIN_, whereas the δ^13^C_EPA_ values were more enriched compared to δ^13^C_LIN_ and δ^13^C_ALA_.

For *Gammarus*, δ^13^C_ALA_ did not differ from δ^13^C_LIN_ in treatments L and M, but in treatment A the δ^13^C_ALA_ values were enriched compared to δ^13^C_LIN_. The δ^13^C_EPA_ values were highly enriched in all treatments. Finally, consistent with patterns in bulk δ^13^C values of these consumers (Figure 3), changes in δ^13^C_LIN_, δ^13^C_ALA_, and δ^13^C_EPA_ values of *Ecdyonurus* and *Gammarus* were highest in treatments A and M, and lowest in treatment L.

## 4 DISCUSSION

In contrast to the assumption of the RCC that upstream consumers depend more on terrestrial than algae‐derived diet, our flume experiment suggests that the shredder *Gammarus* and the grazer *Ecdyonurus* retained a greater proportion of carbon from algae. Our results also demonstrate that both consumers used algae as their main dietary carbon and PUFA source, which is consistent with recent experimental and field studies (Brett et al., 2009; Guo et al., 2018; Lau et al., 2009). Even when fed on leaf litter, both consumers retained more algal carbon, indicating that algae growing on leaves were their preferential dietary resource.

The enriched δ^13^C values in the shredder *Gammarus* and the grazer *Ecdyonurus*, in particular in treatment L, indicate the importance of biofilms attached to leaf litter for stream consumers (Cummins, 1974; Findlay, 2010). Since algal biofilm on leaf litter can account for up to 35% of primary production of streams (Naiman, 1983), algae on leaf litter are a potential food source for invertebrates (Allan & Castillo, 2007; Manning et al., 2015). If invertebrates were exclusively feeding on the biofilm of the leaf litter, the retained carbon would be far more enriched compared to the leaf carbon. Indeed, the δ^13^C values of *Gammarus* and *Ecdyonurus* were very similar when feeding on algae or on the mixed diet, suggesting that both consumers were retaining mainly algal carbon. Similarly, in treatment L, the equally enriched δ^13^C values of *Gammarus* and *Ecdyonurus* suggest that when stream invertebrates are provided with both algae and litter, they preferentially retain algal carbon.

The Chl‐*a* content in the flumes declined over the course of the experiment, as did the grazer biomass. Even low algal biomass can be an important subsidy for stream consumers (Thornton, 1974), although the remaining algal biomass in the present study growing on leaf litter was probably too low to improve the somatic growth of *Ecdyonurus*. Especially before emergence, this grazer needs lots of dietary energy, including lipids and their FA, which consequently leads to a decrease in PUFA content during metamorphosis (Hanson, Cummins, Cargill, & Lowry, 1985). *Ecdyonurus* do not feed after emergence, thus these grazers need to obtain all of their dietary energy for adult life and reproduction prior to emergence. High PUFA demand is also inferred by comparing the initial PUFA contents of *Ecdyonurus* and *Gammarus*. The decreasing PUFA and lipid content in *Ecdyonurus* suggests that algal biomass was too low to provide adequate food in our experiment. In addition, high grazing activity reduces resource biomass, but may increase algae population growth, which can compensate for the algae consumption by consumers (Lamberti & Resh, 1983). However, in our experiment, the algal biomass decrease was not due to feeding pressure, but probably due to the accumulation of sediment particles (field observation). Therefore, it seems that settling sediments reduced algae production, which then limited the somatic growth of consumers. Similar to other emerging insects, mayflies require a substantial amount of stored energy for metamorphosis, egg production, and mating swarms (Winkelmann & Koop, 2007), emphasising the importance of high‐quality food for *Ecdyonurus* to successfully conduct its post‐larval life stage.

Relative to the initial PUFA composition in *Gammarus*, n‐6 PUFA (LIN and ARA) decreased or remained similar, whereas the n‐3 PUFA EPA and ALA increased, indicating selective retention of algal n‐3 PUFA (Guo et al., 2016b) even during decreasing algae supply, as was the case in treatment A. The concurrent decrease in algal supply and n‐6 PUFA in *Gammarus* suggests n‐6 to be less important than n‐3 PUFA for *Gammarus*. Furthermore, the n‐3 PUFA EPA and ALA in *Gammarus* were higher in all the treatments compared to *Gammarus* from the stream. The large contribution of diatoms to the algal community probably accounted for the high n‐3 PUFA content in the consumers since diatoms are rich in EPA (Taipale et al., 2013). The low DHA in *Gammarus* can be attributed to generally low dietary DHA availability, as seen in other studies (Guo et al., 2016b), and/or the ability of *Gammarus* to bioconvert DHA, at low rates, from dietary precursors. However, the long‐chain n‐3 PUFA, in particular EPA and DHA, are not provided by terrestrial leaves (Mills, Mcarthur, Wolfe, Aho, & Rader, 2001), but probably by some algal sources. In general, these results suggest that algae were the most important PUFA provider for *Gammarus*.

Compound‐specific stable isotopes provide evidence that both consumers acquired their FA mostly from algae. Fatty acids had generally 5–15‰ more depleted δ^13^C values than bulk carbon due to isotopic fractionation during lipid synthesis (Gladyshev, Sushchik, Kalachova, & Makhutova, 2012). Relative to these initially depleted values representing natural background, the n‐3 PUFA in consumers were ^13^C‐enriched. The strongest ^13^C‐enrichments occurred in EPA derived from the biofilm. Consumers selected and/or preferentially retained high‐quality resources that already contained this essential PUFA. Consistent with changes in bulk δ^13^C of these consumers, whereby *Ecdyonurus* appear to turn over algal carbon twice as fast as *Gammarus*, EPA turnover in *Ecdyonurus* appeared to be *c. *2× faster than in *Gammarus* as indicated by their difference in algal δ^13^C_EPA_ values between the beginning and the end of this experiment. The isotopically enriched EPA in both consumers from treatment L suggests dietary access of ^13^C‐labelled algae, although periphyton was regularly removed from leaf litter, and indicates that traces of labelled algae‐derived EPA were available and retained in these consumers.

We attribute the high variation in algae δ^13^C values to several factors. First, there is a naturally large natural variation in δ^13^C among different algal taxa (Vuorio, Meili, & Sarvala, 2006) and the various enrichments observed probably reflect different patches of actively photosynthesising algae. Second, different physiological processes among algae or incorporation efficiency of the labelled CO_2_ may have caused the observed high variability in the δ^13^C values. Finally, the different supply of particles in the flume may have *diluted* algae biomass and induced different δ^13^C values. However, even with differing δ^13^C values in algae, our data indicate that the benthic consumers were all enriched in algal carbon.

In conclusion, in contrast to the predictions of the RCC, our study suggests that stream macroinvertebrates, both the shredder *Gammarus* and the grazer *Ecdyonurus*, strongly rely on algae as their main carbon source for somatic tissues, and very little on allochthonous leaf litter. Algae provide more readily available carbon with nutritionally important PUFA to both *Gammarus* and *Ecdyonurus*. However, *Gammarus*, which fed preferentially on leaf litter, nonetheless assimilated FA from attached biofilms; thus, leaf litter itself does not supply PUFA to *Gammarus*, as shown by the discrepancy in FA between leaf litter and consumers. We note that *Gammarus* and other shredders as trophic conveyors of dietary energy, both in terms of quantity and nutritional quality, to higher consumers strongly depend on dietary algal supply. *Ecdyonurus*, referred to as typical grazer, may not be limited to grazing on algae and other easily available diet from rocks, but also from leaf litter. Thus, litter is contributing minimally directly as food source, but serves as an important substrate for algae production (Cummins, 1974; Findlay, 2010; Guo et al., 2016c). Finally, we conclude that both consumers preferentially obtained their carbon and PUFA from algae. Although only two taxa were investigated in this study, it is possible that other grazers and shredders may similarly select and retain their dietary carbon and PUFA from algae.

## Data Availability

Presented data are original data generated for this study. Upon request, data will be made available.
